# Supplementary material for: Meta-Analysis and Gene Set Enrichment Relative to ER Status Reveal Elevated Activity of MYC and E2F in the “Basal” Breast Cancer Subgroup
Source: PLoS One. 2009 Mar 9;4(3):e4710. doi: 10.1371/journal.pone.0004710 (PMC2650420; doi:10.1371/journal.pone.0004710)
Supplement: Dataset S1 — GSEA results from first screen. The file contains a zipped archive of the HTML results of the second GSEA screen. Reports for enriched or depleted datasets may be accessed from the “index.html” link within the my_analysis.GseaPreranked.1219975950625.rpt.zip ZIP archive. Enrichment plots are available for each of the 50 most enriched and depleted gene sets. (13.32 MB ZIP) [file pone.0004710.s001.zip › my_analysis.GseaPreranked.1219975950625.rpt.zip!//CROONQUIST_IL6_STARVE_UP.html]

Details for gene set CROONQUIST\_IL6\_STARVE\_UP[GSEA]

|  || Dataset | zSco.ERneg.to.ERpos\_collapsed |
| Phenotype | NoPhenotypeAvailable |
| Upregulated in class | na\_pos |
| GeneSet | CROONQUIST\_IL6\_STARVE\_UP |
| Enrichment Score (ES) | 0.7436356 |
| Normalized Enrichment Score (NES) | 2.4732568 |
| Nominal p-value | 0.0 |
| FDR q-value | 0.0 |
| FWER p-Value | 0.0 |
Table: GSEA Results Summary

  

Fig 1: Enrichment plot: CROONQUIST\_IL6\_STARVE\_UP      
 Profile of the Running ES Score & Positions of GeneSet Members on the Rank Ordered List

  

| PROBE | GENE SYMBOL | GENE\_TITLE | RANK IN GENE LIST | RANK METRIC SCORE | RUNNING ES | CORE ENRICHMENT || 1 | CDC20 |  |  | 9 | 7.151 | 0.0566 | Yes |
| 2 | FOXM1 |  |  | 21 | 6.760 | 0.1098 | Yes |
| 3 | BUB1 |  |  | 41 | 6.445 | 0.1600 | Yes |
| 4 | CENPA |  |  | 51 | 6.315 | 0.2099 | Yes |
| 5 | CDC45L |  |  | 108 | 5.824 | 0.2523 | Yes |
| 6 | KIF2C |  |  | 124 | 5.703 | 0.2968 | Yes |
| 7 | KIFC1 |  |  | 156 | 5.525 | 0.3387 | Yes |
| 8 | PTTG1 |  |  | 225 | 5.206 | 0.3752 | Yes |
| 9 | CHEK1 |  |  | 253 | 5.088 | 0.4139 | Yes |
| 10 | BUB1B |  |  | 271 | 5.016 | 0.4528 | Yes |
| 11 | OIP5 |  |  | 320 | 4.868 | 0.4881 | Yes |
| 12 | MYBL2 |  |  | 418 | 4.629 | 0.5179 | Yes |
| 13 | CCNB2 |  |  | 431 | 4.589 | 0.5537 | Yes |
| 14 | MAD2L1 |  |  | 477 | 4.446 | 0.5859 | Yes |
| 15 | PLK4 |  |  | 524 | 4.292 | 0.6168 | Yes |
| 16 | SPBC25 |  |  | 606 | 4.126 | 0.6437 | Yes |
| 17 | KIF11 |  |  | 713 | 3.909 | 0.6670 | Yes |
| 18 | CENPE |  |  | 808 | 3.766 | 0.6901 | Yes |
| 19 | GINS1 |  |  | 902 | 3.632 | 0.7121 | Yes |
| 20 | UBE2C |  |  | 994 | 3.489 | 0.7332 | Yes |
| 21 | POLD1 |  |  | 1260 | 3.182 | 0.7387 | Yes |
| 22 | CCNB1 |  |  | 1626 | 2.823 | 0.7339 | Yes |
| 23 | CDKN3 |  |  | 1827 | 2.674 | 0.7402 | Yes |
| 24 | RRM2 |  |  | 2047 | 2.485 | 0.7436 | Yes |
| 25 | TK1 |  |  | 2730 | 2.076 | 0.7089 | No |
| 26 | CSNK1G1 |  |  | 2999 | 1.931 | 0.7042 | No |
| 27 | WEE1 |  |  | 3048 | 1.909 | 0.7159 | No |
| 28 | CDC6 |  |  | 3185 | 1.847 | 0.7204 | No |
| 29 | TOP2A |  |  | 3735 | 1.610 | 0.6920 | No |
| 30 | SPP1 |  |  | 4426 | 1.346 | 0.6508 | No |
| 31 | LIG1 |  |  | 4659 | 1.266 | 0.6435 | No |
| 32 | ZWINT |  |  | 5940 | 0.834 | 0.5539 | No |
| 33 | KIF22 |  |  | 8725 | -0.180 | 0.3458 | No |
Table: GSEA details [plain text format]

  

Fig 2: CROONQUIST\_IL6\_STARVE\_UP: Random ES distribution      
 Gene set null distribution of ES for **CROONQUIST\_IL6\_STARVE\_UP**
